# Food deprivation alters reproductive performance of biocontrol agent *Hadronotus pennsylvanicus*

**DOI:** 10.1038/s41598-022-11322-5

**Published:** 2022-05-03

**Authors:** Robert K. Straser, Houston Wilson

**Affiliations:** Department of Entomology, University of California, Riverside, Riverside, CA 92521 USA

**Keywords:** Agroecology, Animal behaviour, Entomology

## Abstract

Diet can influence parasitoid reproductive performance, and therefore, the efficacy of biocontrol programs. We evaluated the influence of food deprivation on the reproductive fitness and behavior of the egg parasitoid *Hadronotus pennsylvanicus* (Hymenoptera: Scelionidae), a prospective biocontrol agent for *Leptoglossus zonatus* (Heteroptera: Coreidae). Newly emerged female parasitoids were mated and provided host eggs every other day while being provisioned with various honey diet regimes or a consistent supply of water. When given frequent access to a honey diet, female parasitoids lived significantly longer and parasitized more host eggs compared to the water-fed controls. Once depleted of mature eggs, females with frequent access to honey also contributed to greater non-reproductive host mortality. Furthermore, behavioral assays demonstrated that water-fed females spent less time interacting with host eggs and tended to more frequently divert from oviposition behavior. While there was no difference in the average duration until first oviposition between individuals assigned to different diet treatments, increased frequency of honey feeding was associated with more frequent and longer duration of oviposition. The positive effect of honey feeding on the reproductive performance of biocontrol agent *H. pennsylvanicus* suggests that performance of this parasitoid under field conditions could be enhanced through the provision of similar carbohydrate resources, such as flowering summer cover crops.

## Introduction

Understanding the influence of diet on parasitoid reproduction is critical for the development of effective biocontrol programs. Access to suitable food sources has shown to significantly improve both the survival and lifetime reproductive fitness of various parasitoids^1,2^. In the field, adult parasitoids obtain food through either floral (nectar and pollen), extra-floral (nectaries), or non-floral (honeydew and host feeding) resources^3–6^. These diets provide vital nutrients to fuel bodily maintenance and physiological energy expenditure, enhancing the dispersal capacity and fertility of adult wasps^5,7^. In field settings, the quality and availability of these resources can vary widely in time and space. Periods of insufficient access to sources of carbohydrate-rich food can result in decreased survival, fecundity, and altered behavior of female parasitoids, all of which can negatively impact their biocontrol potential^1,2^.

In order to maximize lifetime reproduction, female parasitoids must balance time limitations, egg limitations, and the costs of oviposition^8–10^. For synovigenic parasitoids, egg limitation can occur when female egg loads are depleted at a faster rate than they are able to produce. When host resources are abundant, synovigenic parasitoids risk depleting their cache of mature eggs before exhausting their supply of available host eggs^11^. Furthermore, insufficient access to suitable diet may reduce their ability to replenish egg loads in response to higher host densities, since the availability of a carbohydrate-rich diet has been shown to enhance the oviposition behavior and parasitism rates for various parasitoids^2^. Similarly, feeding frequency has also been shown to be vital for parasitoid survival and reproductive fitness^2^. Increased abundance of nutrient rich food sources has been associated with an increase in searching ability, enhanced mobility and sensitivity to olfactory cues, as well as host acceptance^12,13^. In choice assays, satiated wasps preferred a host stimuli over a food resource, whereas starved wasps exhibited no preference, taking more time to initiate searching behavior and to locate a cue^14–16^. Such patterns in parasitoid behavior may be exacerbated during periods of low food resource availability and extended starvation.

*Hadronotus* (= *Gryon*^17^) *pennsylvanicus* (Ashmead) (Hymenoptera: Scelionidae) is a generalist synovigenic egg parasitoid, known to attack the leaffooted plant bug, *Leptoglossus zonatus* (Dallas) (Heteroptera: Coreidae)^18^ along with several other coreid pests including *L. phyllopus* (L.)^19^, *L. australis* Fabricius^20^, *L. occidentalis* Heidemann^21–23^, *Anasa tristis* (DeGeer)^24,25^, and *A. armigera* Say^26^. In California, U.S.A., *L. zonatus* has become an increasingly problematic pest of almonds (*Prunus dulcis* L.) and pistachios (*Pistacia vera* L.)^27–29^. Feeding from *L. zonatus* has the potential to cause significant damage to these nut crops and can facilitate such fungal contaminations as stigmatomycosis and Botryosphaeria, all of which lowers crop yield and quality^30–36^. Under laboratory conditions, *H. pennsylvanicus* exhibits suitable demographic and reproductive traits suggesting its potential use as a biocontrol agent for *L. zonatus*^18^. However, access to a carbohydrate-rich diet is likely critical to maximize lifetime fitness of *H. pennsylvanicus*. Previous studies demonstrated that females deprived of a honey-water diet died shortly after eclosion, resulting in significantly fewer offspring over their short lifetime^18,37–39^. Furthermore, egg limitation appears to be a constraint on lifetime fecundity of female *H. pennsylvanicus*^18,26,38^. Under continuous reproductive opportunity, the proportion of parasitoid-induced aborted eggs increased with female age, ultimately accounting for the majority of total host egg mortality^18^. Since *H. pennsylvanicus* likely does not feed on their host^40^, such non-reproductive host mortality may facilitate additional pest control in the absence of mature eggs. However, little is known about the extent of non-reproductive mortality, much less the rate at which *H. pennsylvanicus* are capable of replenishing egg loads when provided sufficient access to a nutritious diet.

Improved knowledge on the role of diet on the reproductive performance of *H. pennsylvanicus* would allow a better understanding of where and under what conditions resource provisioning could be used to improve parasitoid field performance, and by way of that, biocontrol of *L. zonatus*. Here, a series of experiments under controlled laboratory settings were carried out to evaluate the impact of food deprivation on *H. pennsylvanicus* demographic parameters, reproductive traits, non-reproductive host mortality, and female reproductive behavior. This framework may provide new insight into the role of diet acquisition on the efficacy of biocontrol programs.

## Results

### Effects of food deprivation on parasitoid demographic & reproductive traits

Diet availability significantly influenced the longevity and lifetime fecundity of female *H. pennsylvanicus* ($\chi^{2}$ = 152.27, df = 4, *P* < 0.001 for longevity; $\chi^{2}$ = 35.26, df = 4, *P* < 0.001 for fecundity) (Table 1). Similarly, diet availability influenced the duration of ovipositional and post-reproductive periods for females ($\chi^{2}$ = 73.75, df = 4, *P* < 0.001 for ovipositional period; $\chi^{2}$ = 94.90, df = 4, *P* < 0.001 for post-reproductive period). Females fed a honey-water diet two or three times per week lived significantly longer and exhibited greater ovipositional and post-reproductive periods throughout their lifetime than females fed honey-water once per week, every other week, or a water-only diet. When provided host eggs, females fed a honey-water diet twice per week lived 38.80 ± 2.79 days, while females fed three times per week lived 41.60 ± 2.75 days. Water-fed females provided host eggs only lived 2.40 ± 0.31 days. Females fed a honey-water diet three times per week maintained the greatest lifetime fecundity (48.10 ± 7.07 offspring), while females fed honey-water less than twice per week or fed water exhibited fewer offspring over their lifespan. However, diet availability did not influence the average female sex ratio of offspring over a females lifetime.

**Table 1:** Reproductive parameters (mean ± SE) of Hadronotus pennsylvanicus reared on Leptoglossus zonatus eggs every other day at 25 °C ± 1, 75 ± 5% RH and 16:8 L:D, fed a honey-water diet once (× 1), twice (× 2), three times (× 3) per week, once every other week (× 0.5), or fed a water diet (× 0).

Reproductive parameters	*x*0	*x*0.5	*x*1	*x*2	*x*3
Adult female longevity (days)	2.40 ± 0.31 a	8.20 ± 0.95 b	9.20 ± 0.70 b	38.80 ± 2.79 c	41.60 ± 2.75 c
Ovipositional period (days)	1.20 ± 0.33 a	4.40 ± 0.60 b	3.20 ± 0.55 b	10.40 ± 1.19 c	11.20 ± 1.09 c
Post-reproductive period (days)	1.20 ± 0.29 a	3.80 ± 0.92 b	6.00 ± 1.03 b	28.40 ± 3.38 c	30.40 ± 2.96 c
Total progeny (offspring/female)	8.90 ± 2.49 a	22.10 ± 3.57 b	17.00 ± 1.98 b	36.10 ± 5.52 bc	48.10 ± 7.07 c
Total female progeny (females/female)	7.60 ± 1.98 a	20.40 ± 3.42 b	14.60 ± 1.74 b	31.20 ± 5.05 bc	42.10 ± 5.93 c
Total male progeny (males/female)	1.30 ± 0.72 a	1.70 ± 0.47 ab	2.40 ± 0.67 abc	4.90 ± 0.87 bc	6.00 ± 1.80 c
Sex ratio (% female offspring)	89.57 ± 3.95 a	94.10 ± 1.34 a	86.10 ± 4.32 a	85.30 ± 1.75 a	88.60 ± 2.18 a

Parameters with different letters indicate significant differences across the different treatments (*P* < 0.05; GLM and Tukey post hoc test).

**Figure 1:**
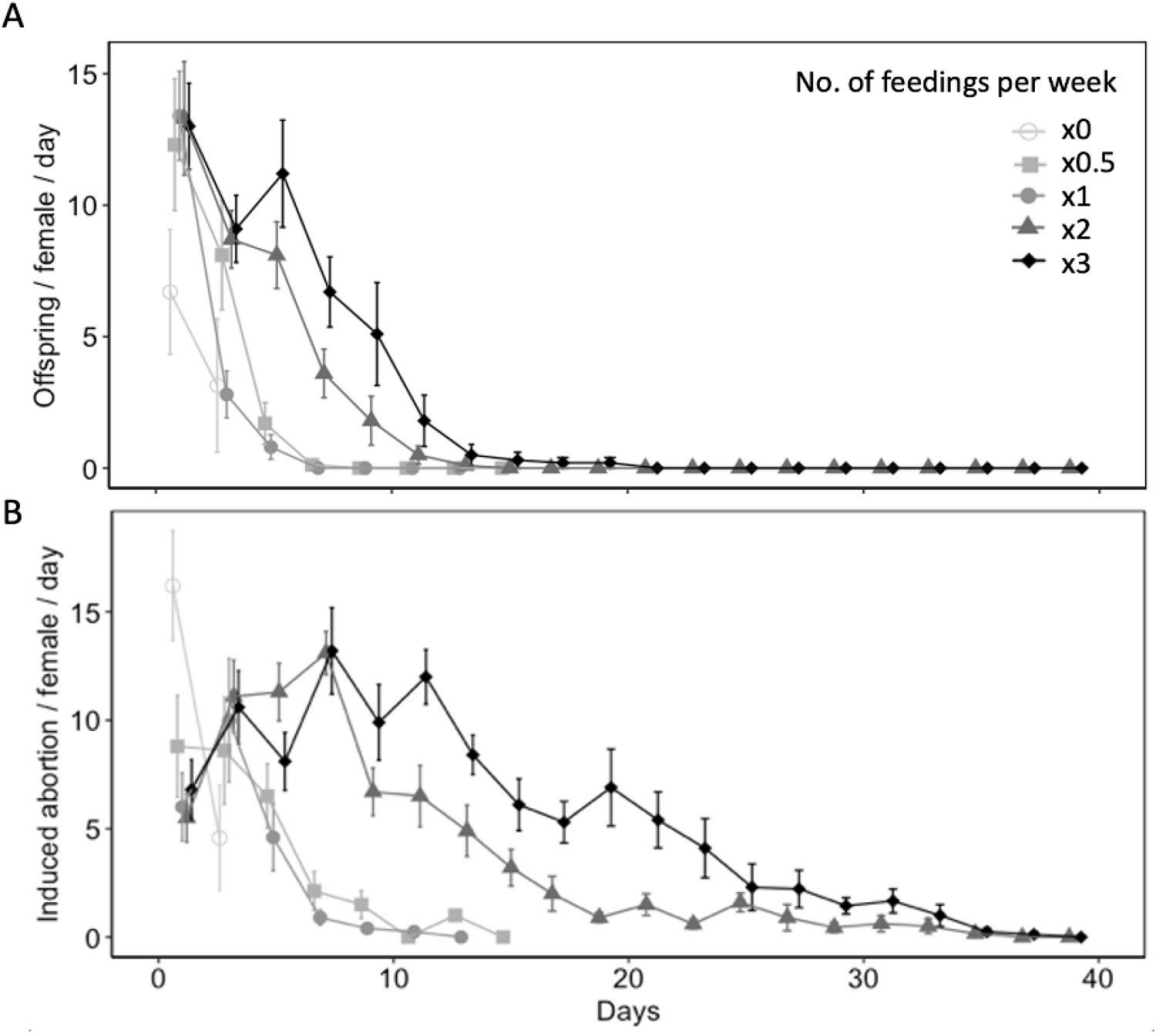
Age specific fecundity (**A**) and induced egg abortions on host (**B**) (mean ± SE) of *Hadronotus pennsylvanicus* when provided *Leptoglossus zonatus* host eggs and fed honey-water diet once (× 1), twice (× 2), or three (× 3) times per week, every other week (× 0.5), or fed a water-only diet (× 0).

Diet availability significantly influenced reproductive and non-reproductive host mortality ($\chi^{2}$ = 35.26, df = 4, *P* < 0.001 for offspring produced per female per day; $\chi^{2}$ = 30.45, df = 4, *P* < 0.001 for induced abortions per female per day) (Fig. 1A). All parasitism occurred within first 15 days of the females lifetime, with the highest mean parasitism rates occurring on day 1 of study. On day 1, water-fed females produced fewer offspring (6.70 ± 2.38) than females provided honey-water diet once (13.40 ± 1.69) or three times per week (13.00 ± 1.64) (z = 2.84, *P* < 0.05 for × *1*; z = 2.74, *P* < 0.05 for × 3), however was not significantly different than females fed honey-water twice (13.30 ± 2.16) or every other week (12.30 ± 2.50) (z = 2.66, *P* = 0.06 for × *1*; z = 2.29, *P* = 0.15 for × *0.5*). Fecundity showed relatively consistent declines over the first 3 weeks for all diet treatments. However, females provided honey-water twice or three times per week maintained higher fecundity rates on days 5 (× 2, 8.10 ± 1.27; × 3, 11.20 ± 2.04), 7 (× 2, 3.60 ± 0.92; × 3, 6.70 ± 1.33), and 9 (× 2, 1.80 ± 0.93; × 3, 5.10 ± 1.96) in comparison to individuals fed once per week, every other week, or a water-only diet.

### Effects of food deprivation on non-reproductive host mortality

There was a significantly greater proportion of aborted host eggs observed when *L. zonatus* eggs were exposed to a female wasp than those unexposed ($\chi^{2}$ = 31.62, df = 1, *P* < 0.001). In the absence of a parasitoid, the average proportion of eggs determined aborted was 0.70 ± 0.26. Diet availability also influenced non-reproductive host mortality over the course of a females lifetime ($\chi^{2}$ = 146.17, df = 4, *P* < 0.001 for induced egg abortions per female per day) (Fig. 1B). Water-fed females exhibited greater rates of induced host abortion (16.20 ± 2.52) on day 1, however this trend declined to 4.57 ± 2.42 on day 3 of the study. When females were fed a honey-water diet, individuals fed twice or three times per week maintained higher rates of induced abortions than individuals fed honey-water once per week or every other week (z = 4.10, *P* < 0.01 for × *2*; z = 5.52, *P* < 0.01 for × 3). For honey-fed females provided diet twice and three times per week, female age had a significant influence on the proportion of induced abortions (z = 6.19, *P* < 0.01). Here, females fed a honey-water diet three times per week exhibited a greater number of induced abortions than wasps fed diet twice per week from day 11 (z = 2.92, *P* < 0.01) through day 23 (z = 3.42, *P* < 0.01) of the study.

### Effects of food deprivation on parasitism behavior

**Figure 2:**
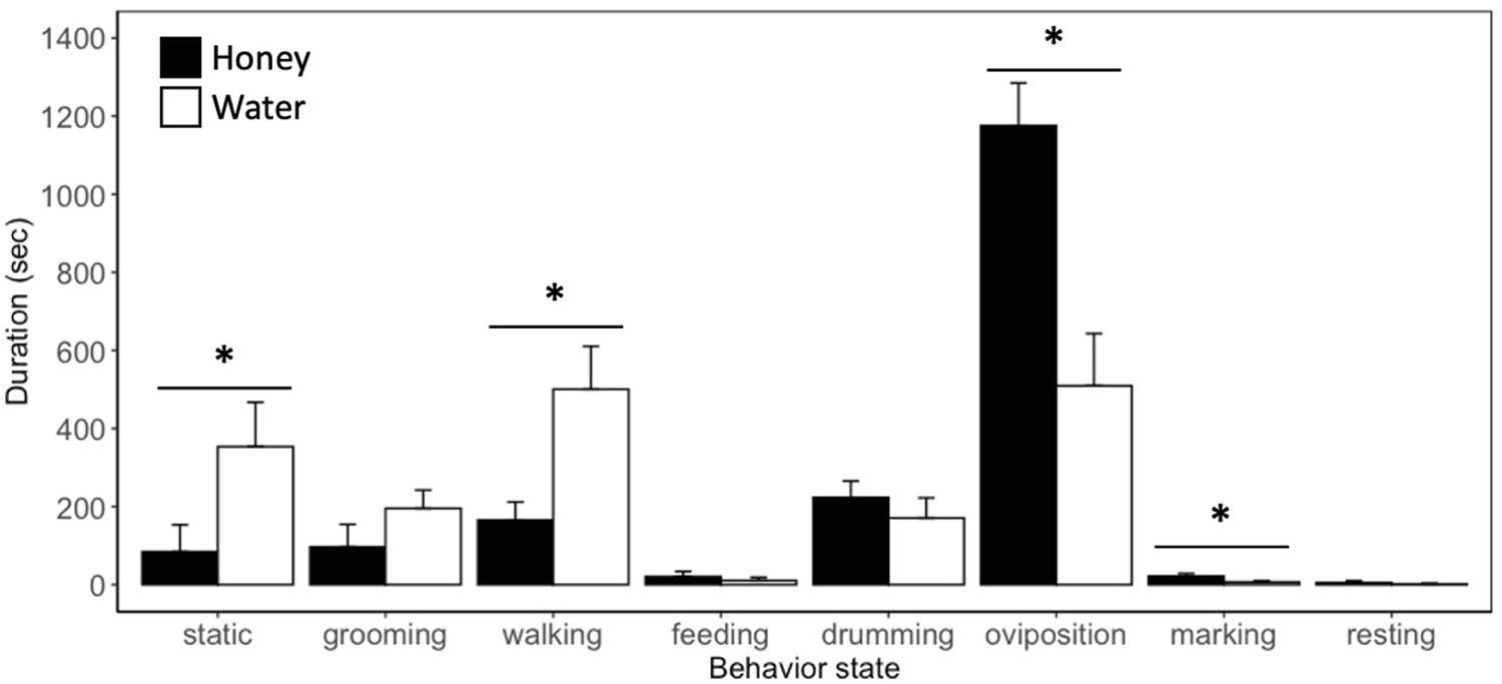
Duration (mean + SE) spent within each behavior state in 30 min period by *H. pennsylvanicus* female when provided *L. zonatus* host eggs and fed a honey-water (honey) or water diet (water). Statistical significance between treatments indicated by asterisk (*P* < 0.05; GLM and likelihood ratio chi-squared test).

**Figure 3:**
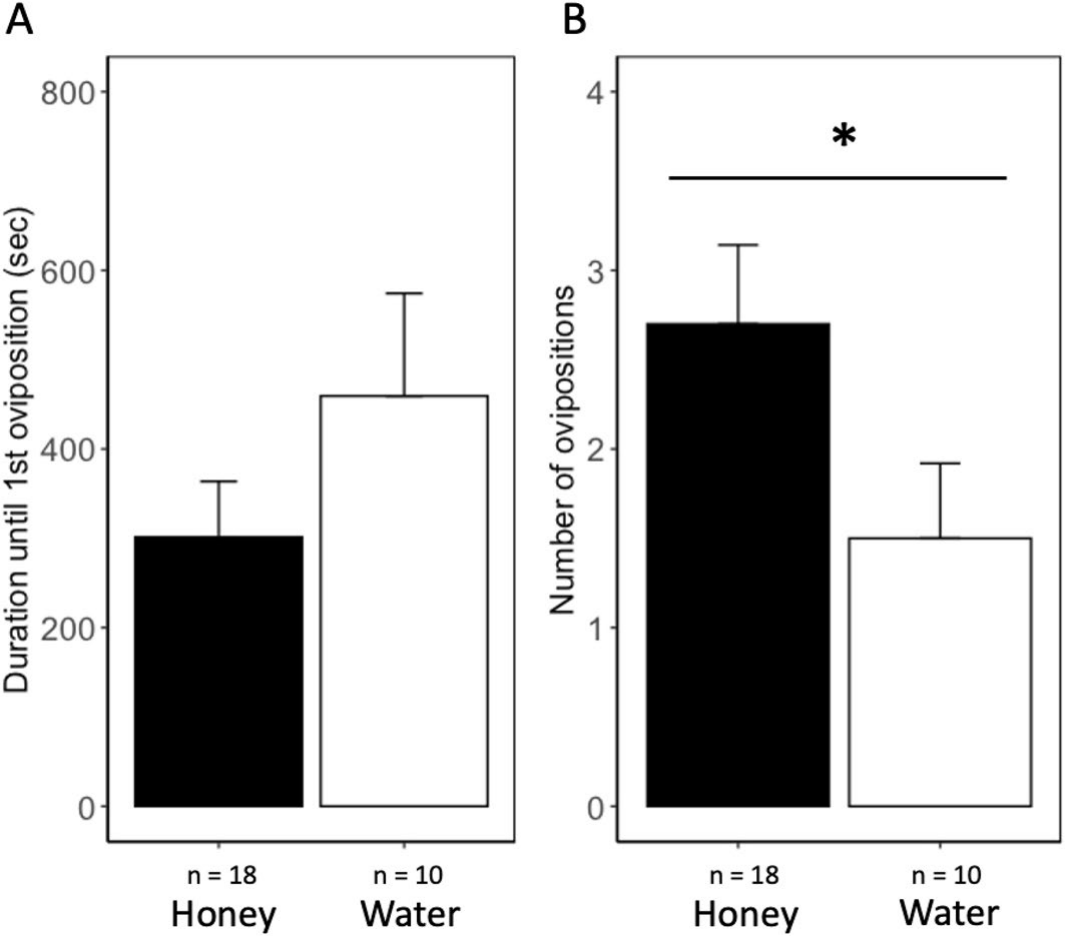
Duration until the first oviposition on host (**A**) and the number of ovipositions (**B**) (mean + SE) within 30 min period by *H. pennsylvanicus* female when provided *L. zonatus* host eggs and fed a honey-water (honey) or water-only diet (water). Statistical significance between treatments indicated by asterisk (*P* < 0.05) (GLM and likelihood ratio chi-squared test).

Diet influenced the duration parasitoids spend in behavioral states (Fig. 2). During the 30 min observation period, honey-fed females spent on average 74.82 ± 8.51% of their time interacting with host eggs, whereas water-fed females spend 37.09 ± 10.16% of their time. Water-fed females spent significantly more time walking ($\chi^{2}$ = 7.56, df = 1, *P* < 0.01) or remaining static ($\chi^{2}$ = 4.11, df = 1, *P* < 0.05) in the arena than honey-water fed females. However, diet did not influence the amount of time females spent grooming or feeding from provided diet. When interacting with host eggs, honey-water fed females spent more time ovipositing in eggs than water-fed individuals ($\chi^{2}$ = 13.18, df = 1, *P* < 0.001). However, there was no significant difference in the duration of time spent drumming, marking, or resting between honey- and water-fed individuals. While there was no difference in the average duration until females began ovipositing (Fig. 3A), 50% of water-fed females failed to oviposit on host eggs, whereas only 10% of honey-water fed females failed to oviposit. Of the individuals that exhibited oviposition behavior, honey-water fed females oviposited more frequently than females fed a water diet ($\chi^{2}$ = 3.88, df = 1, *P* < 0.05) (Fig. 3B). Females fed honey-water oviposited 2.7 ± 0.44 times within a 30 min period while females fed water diet oviposited 1.50 ± 0.42 times.

**Figure 4:**
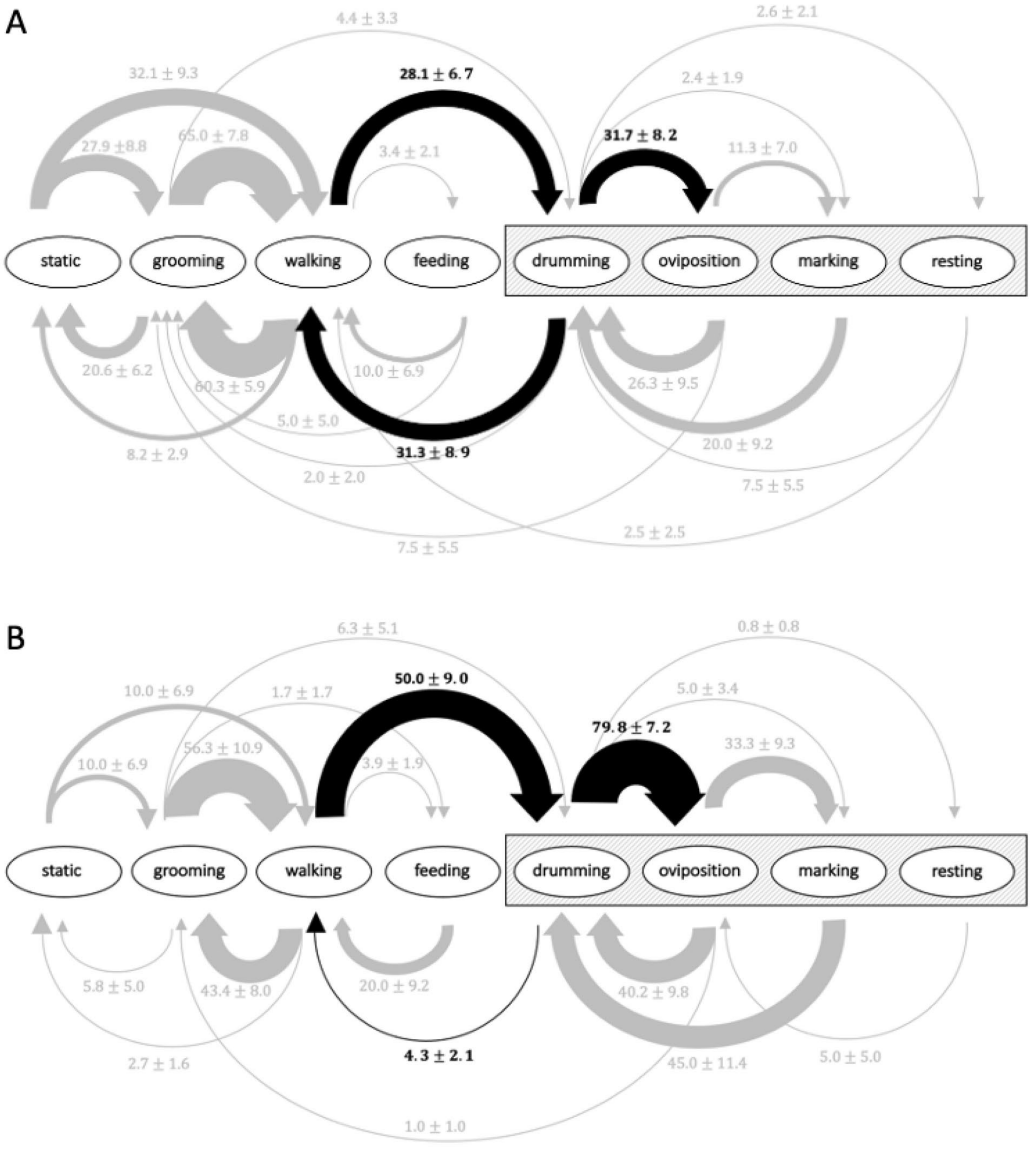
Ethogram of the proportion of transitions between behavior states (mean ± SE) within a 30 min period by *H. pennsylvanicus* female when provided *L. zonatus* host eggs and fed a water (**A**) or honey-water diet (**B**). Grey box highlights behaviors in which parasitoid is interacting with host eggs. Line thickness indicates relative frequency of transition. Black arrows highlight behaviors where a statistical difference was observed between the two treatments (*P* < 0.05; GLM and likelihood ratio chi-squared test).

Adult diet influenced the frequency of behavioral transitions in female *H. pennsylvanicus* (Fig. 4). Females fed a honey-water diet more frequently transitioned from walking to drumming behavior on the host ($\chi^{2}$ = 5.06, df = 1, *P* < 0.05). Here, 50.00 ± 9.00% of honey-water fed females transitioned from walking to drumming behavior, whereas only 28.10 ± 6.70% of water fed females made the transition. Once interacting with host eggs, honey-water fed individuals more frequently transitioned from drumming to oviposition (79.80 ± 7.20% for honey-water fed, 31.70 ± 8.20% for water fed) ($\chi^{2}$ = 8.63, df = 1, *P* < 0.01). Furthermore, water-fed females more frequently transitioned from drumming host eggs to walking behavior (31.30 ± 8.90% for water fed, 4.30 ± 2.10% for honey-water fed) ($\chi^{2}$ = 7.65, df = 1, *P* < 0.01), aborting parasitism behavior on the host. There was no significant difference in the frequency of all other behavior state transitions between honey-fed and water-fed females.

## Discussion

Access to carbohydrate-rich food sources has shown to bolster the longevity, dispersal, and fecundity of natural enemies, and therefore enhance the efficacy of biocontrol^3,5,6^. During periods of food deprivation, parasitoids may spend more time foraging, reabsorbing eggs to extend longevity, or maturing eggs more rapidly in order to maximize reproductive fitness^10,41–43^. The aim of this study was to assess how inadequate access to diet impacts the fitness and reproductive performance of the egg parasitoid *H. pennsylvanicus*, a key biocontrol agent for the tree nut pest *L. zonatus*. Under laboratory conditions, we found support for the theory that food deprivation negatively impacts the longevity, lifetime fecundity, and reproductive behavior of parasitoids^1,2^.

When provided frequent access to a carbohydrate-rich diet, female *H. pennsylvanicus* produced more offspring while maintaining extended ovipositional periods. Alternatively, females fed < 2 times per week exhibited a drastic decline in offspring production early in their lifetime. Given *H. pennsylvanicus* does not show evidence of egg resorption^40^, this may indicate that females are capable of maturing eggs only when provided sufficient access to diet. Previous studies have demonstrated that consistent access to food, rather than longer feeding intervals, provides greater benefits to adult longevity^44^. Wu et al.^45^ found parasitoids provided with continuous access to a sugar diet produced significantly more offspring than those provided fewer opportunities to feed on a sugar diet. Theory suggests that insufficient access to a suitable diet reduces the ability of synovogenic parasitoids to replenish egg loads^2^. Our finding suggest that during period of low food resource availability, *H. pennsylvanicus* may be unable to produce egg loads at a sufficient rate to suppress high pest densities in cropping systems. While we did not observe female egg load, future research that documents egg count over the course of a female lifetime would illuminate the link between egg maturation and diet.

The sex ratio of progeny of parasitoids can be influenced by several biotic and abiotic factors, such as parental age, host quality, female population density, and environmental temperature^46^. While Berndt et al.^47^ found that food deprivation in female wasps can lead to male-biased progeny, parasitoids respond to external pressures differently^2^. However, we found that frequent access to honey-water did not influence the female sex ratio of *H. pennsylvanicus* progeny over lifetime of a female. Instead, *H. pennsylvanicus* appears to maintain consistently high female skewed progeny despite ovipositing female age and host age^18,48^. These outcomes vary between host species^24,38^. Previous studies have found that such trends in sex ratio are due to sperm depletion in females despite being held under conditions of continuous copulation opportunity^38^. However, little is known on how sperm depletion may influence the reproductive performance of egg parasitoids, and this requires further evaluation. While parasitoid fecundity is ultimately dependent on host availability, maintaining a high female-skewed sex ratio in progeny despite food availability would likely benefit parasitoid population growth rates in field settings.

Egg parasitoids provide biocontrol by killing the host egg. Oviposition of a parasitoid egg into the host egg results in host egg mortality and reproduction of the parastioid (i.e. reproductive mortality). However, non-reproductive host mortality also plays an important, and often understated role in biocontrol programs for insect pests^49^. Previous studies have found that when *H. pennsylvanicus* was provided honey-water ad libitum, non-reproductive host mortality accounted for > 50% of the total host mortality observed within the first 2 weeks of a females life^18^. Furthermore, researchers found that the proportion of parasitoid-induced aborted eggs increased with female *H. pennsylvanicus* age, ultimately accounting for the majority of total host egg mortality^18^. In this study, we found that the frequency in which diet was provided influenced parasitoid-induced host abortion rates over the lifetime of a female wasp. Though there was large variation between diet treatment groups, initial feeding following parasitoid emergence appeared to impact the proportion of non-reproductive host egg mortality thereafter. Starved females exhibited their highest rates of induced host abortions on day 1 of the study, whereas wasps fed ≥ 2 times per week maintained higher rates of induced abortions as successful production of progeny began to decline. Our findings may suggest that during extended periods of food deprivation, *H. pennsylvanicus* females may maintain parasitism activity despite reaching potential egg maturation limits. Such trends in parasitoid induced abortions may help elucidate the role that non-reproductive host mortality may play in applied biocontrol programs for *L. zonatus*. However, further research is needed to better understand how diet constraints may impact the parasitism rates by *H. pennsylvanicus* females, and furthermore, the relative proportion of insect-induced host egg abortions resulting in the suppression of *L. zonatus* in field settings.

Female parasitoids primarily allocate energy to locate a host or forage for food^2^. If their diet does not fulfill their nutritional requirements, they may not be able to locate food or hosts^1^. While in general, starved parasitoids tend to show preference in locating a food resource rather than a host, these trends can often vary and outcomes may be species specific^50–54^. Previous studies have demonstrated that food deprivation can enhance the oviposition behavior of egg parasitoids, resulting in increased female reproductive fitness^55^. For instance, Takano et al.^55^ found that starved females of egg parasitoid *Paratelenomus saccharalis* (Dodd) (Hymenoptera: Platygastridae) oviposited quicker and more frequently on host eggs than satiated females. These findings would suggest parasitoids would exhibit preference to reproduce as soon as possible when under conditions of potentially high mortality for adults. However, our results contradict with these findings. We found that honey-fed females spent more time interacting with host eggs, and more commonly engaged in parasitism behaviors. Furthermore, of the water-fed *H. pennsylvanicus* females that engaged in drumming behavior, an average 31% of those individuals abandoned the host eggs prior to oviposition and did not complete parasitism. These findings would suggest that *H. pennsylvanicus* may seek suitable carbohydrate-rich diets over opportunities for reproduction. However, it is important to note that *H. pennsylvanicus* parasitism behavior can vary between host species. Cornelius et al.^39^ found that when provided continuous access to honey diet, *H. pennsylvanicus* maintained increased rates of parasitism on *A. tristis* egg masses, however diet did not impact parasitism of *A. armigera* egg masses. Female wasps also spend the same amount of time probing egg masses of the two host species, however they spend significantly more time drilling *A. tristis* eggs than *A. armigera* eggs^39^. Little is known about how individual variation within species and between-species variation may influence parasitoid behavior in responses to food deprivation.

Under field conditions, the availability of carbohydrate-rich resources can vary widely. Parasitoids may experience periods with insufficient access to a suitable diet, which can negatively impact their reproductive fitness and the efficacy of biocontrol programs^1^. A nuanced understanding of the influence of food deprivation on host parasitism may lead to improved strategies to enhance biocontrol within integrated pest management programs, such as the optimization of floral resource provisioning^56^. The spatial and temporal arrangement of habitat resources necessary to provide reliable biocontrol services in a given agroecosystem is contingent on a variety of ecological and agronomic factors, including natural enemy feeding frequency requirements^1^, as demonstrated here. Findings from this study suggest that floral resource provisioning in tree nut orchards to enhance *H. pennsylvanicus* populations should be designed to maximize and increase parasitoid feeding frequency. Here, there remains a need to better understand the phenology of both pest and parasitoid in relation to the floral provisions, how *H. pennsylvanicus* populations may respond to extended periods of low food resource availability, and furthermore, when and where resource provisioning may bolster biocontrol services in applied settings. For the latter, it will be important to characterize potential energetic tradeoffs between foraging and host location efforts and behavior under different resource availability scenarios.

## Methods

### Insect colonies

Laboratory insect colonies were reared from specimens collected in 2017 from Fresno County, California, USA. *Leptoglossus zonatus* were housed in cages (60 × 60 × 60 cm, 680 µm mesh, BugDORM®, Taiwan) that contained a 2.36 L potted juniper (*Juniperus* sp. L.) plant along with a diet of fresh zucchini (*Cucurbita pepo* L.), green beans (*Phaseolus vulgaris* L.) and organic raw sunflower seeds (*Helianthus annuus* L.) that was refreshed weekly. Wooden skewers (25.4 cm × 3 mm) were used as oviposition substrate. *Hadronotus pennsylvanicus* colonies were reared from parasitized sentinel *L. zonatus* egg clusters from pistachio orchard in Parlier, California, USA, and refreshed with wild strain specimens in 2019 and 2020 from pistachio orchards in Fresno County, USA. Parasitoids were housed in 50 ml plastic tubes (Falcon Conical Centrifuge Tubes, Fisher Scientific®) sealed at the open end with 250 µm mesh, and fed honey-water (1:1) (Organic Honey, Wholesome®) ad libitum. *Hadronotus pennsylvanicus* was identified to species using Masner^57^ and taxonomic revisions in Talamas et al.^17^. All bioassays were conducted in laboratory conditions of 25 °C ± 1, 75 ± 5% RH and 16:8 L:D photoperiod.

### Effects of food deprivation on parasitoid demographic and reproductive traits

To investigate the influence frequency of food provisions has on *H. pennsylvanicus* reproductive output, newly emerged (< 24 h) males and females were housed in mating pairs in 15 ml plastic tubes (Falcon Conical Centrifuge Tubes, Fisher Scientific®) sealed with a 250 µm mesh cap. Each mating pair was provided a honey-water diet (1:1) either once (× 1), twice (× 2), three (× 3) times per week, every other week (× 0.5), or fed a water diet (× 0) three times per week (n = 15 per treatment).

Honey-water was used to standardize the nutrient content of the diet available to wasps throughout the study. A set of 25, fresh (< 72 h old) *L. zonatus* eggs were provisioned every other day for oviposition until the female died. In order to maintain copulation potential throughout the study, a new male specimen was introduced to the enclosure to replace any expired individuals within 24 h. Following 24 h exposure to the parasitoids, *L. zonatus* eggs were removed and observed daily for the emergence of parasitoids or *L. zonatus* nymphs. After 30 days, eggs that did not yield a nymph or adult wasp were dissected to examine for the presence of a dead *L. zonatus*, dead *H. pennsylvanicus*, or determined aborted should neither be discernable.

### Effects of food deprivation on non-reproductive host mortality

To test the influence of food accessibility on non-reproductive host mortality, the number of aborted host eggs was compared between diet frequency treatments. To assess the influence parasitoid presence has on host egg abortion, 15 fresh (< 72 h old) unexposed *L. zonatus* egg clusters containing 25 eggs each were isolated simultaneously with day 1 replicates of fecundity assays. Similarly, egg clusters were housed in 15 ml plastic tubes closed with a 250 µm mesh and stored under controlled laboratory conditions for 30 days. Egg clusters were observed for the emergence of *L. zonatus* nymphs and all unemerged eggs remaining were dissected to test for presence of dead nymphs or identified as aborted if contents were indiscernible. Observations from fecundity assay dissections did not account for the presence or absence of a parasitoid meconium. The number and frequency of host-eggs provided to parasitoids were selected based on the maximum parasitism observed within 24 h, and previous findings that short term host deprivation does not influence parasitism rates^18,40,48^.

### Effects of food deprivation on parasitism behavior

To assess the influence of diet on parasitism behavior, newly emerged (< 24 h) females were randomly selected and fed either a honey-water diet (1:1) (n = 20) or distilled water (n = 20). Newly emerged female *H. pennsylvanicus* individuals were housed individually with a newly emerged male for 24 h prior to observation. After 24 h, females were removed and placed individually in a 54 × 14 mm Petri dish arena containing a set of 25 fresh (< 72 h old) *L. zonatus* eggs and 3 drops of the designated diet treatment. Parasitoid behavior was recorded using a Dino-Lite Digital Microscope (5MP Edge AM7915MZT, AnMo Electronics Co., Taiwan) for 30 min following the placement of the female in the center of the arena. All behavior assays were conducted between 08:00 and 12:00 during peak parasitism activity^58^. Following Wiedemann et al.^59^, behavior was recorded as discrete states: static (remaining motionless or inactive following introduction), walking (host searching, walking or exploring arena), drumming (antennating egg clusters rapidly), oviposition (inserting ovipositor into host-egg for egg laying), marking (scratching ovipositor on egg surface to mark host-egg), grooming (cleaning body parts using legs or mouth), resting (remaining motionless on egg cluster), feeding (drinking from diet droplet). Behavioral observations were analyzed using Behavioral Observation Research Interactive Software (BORIS)^60^ to quantify the number of ovipositions, duration spent on each behavior state, and the frequency of transitions between behavior states.

### Data analysis

Data on parasitoid longevity and fecundity were used to calculate biological and reproductive traits of female *H. pennsylvanicus* on host *L. zonatus* under each diet frequency treatment. The effect of diet access frequency on parasitoid longevity, fecundity, and reproductive behavior were evaluated with generalized linear mixed models (GLMM). A Gamma error distribution and an identity link function were used to assess parasitoid longevity, while a Poisson error and log-link function were used to assess ovipositional period, post reproduction period and total progeny produced, and a binomial error distribution used with an identity link function to assess progeny sex ratios. The effect of diet access frequency on number of ovipositions, duration spent in each behavior state, and frequency of behavioral transitions were assessed using a Gaussian distribution and identity link function. Analyses were carried out with R version 3.6.1^61^. GLMM analyses were conducted using the “glmer” function in the “lme4” package^62^. Fixed effects were evaluated through model comparisons using likelihood ratio test via the “drop1” function. When multilevel variables were found to be significant, means were separated with a post hoc Tukey test using the “glht” function in the “multcomp” package^63^. All data are presented as mean ± SE.

## Research involving plants

All plant materials used were classified as of least concern and readily sourced through public purchase, therefore in accordance with IUCN Policy Statement on Research Involving Species at Risk of Extinction and Convention on the Trade in Endangered Species of Wild Fauna and Flora.

## Data Availability

The datasets used and/or analyzed during the current study available from the corresponding autho**r** on request.
